# Supplementary material for: A fault-intrusion-tolerant system and deadline-aware algorithm for scheduling scientific workflow in the cloud
Source: PeerJ Comput Sci. 2021 Nov 2;7:e747. doi: 10.7717/peerj-cs.747 (PMC8576554; doi:10.7717/peerj-cs.747)
Supplement: Supplemental Information 6 [file peerj-cs-07-747-s006.zip › Myproject1.1/docs/org/cloudbus/cloudsim/Storage.html]

Storage


JavaScript is disabled on your browser.


- Overview
- Package
- Class
- Use
- Tree
- Deprecated
- Index
- Help

- Prev Class
- Next Class

- Frames
- No Frames

- All Classes

- Summary:
- Nested |
- Field |
- Constr |
- Method

- Detail:
- Field |
- Constr |
- Method


org.cloudbus.cloudsim

## Interface Storage

- All Known Implementing Classes:
  :   ClusterStorage, HarddriveStorage, SanStorage

  ---

    

  ```
  public interface Storage
  ```

  An interface which defines the desired functionality of a storage system in a Data Cloud. The
  classes that implement this interface should simulate the characteristics of different storage
  systems by setting the capacity of the storage and the maximum transfer rate. The transfer rate
  defines the time required to execute some common operations on the storage, e.g. storing a file,
  getting a file and deleting a file.

  Since:
  :   CloudSim Toolkit 1.0

- - ### Method Summary

    Methods

    | Modifier and Type | Method and Description |
    | --- | --- |
    | `double` | `addFile(File file)` Adds a file to the storage. |
    | `double` | `addFile(java.util.List<File> list)` Adds a set of files to the storage. |
    | `double` | `addReservedFile(File file)` Adds a file for which the space has already been reserved. |
    | `boolean` | `contains(File file)` Checks whether a file is stored in the storage or not. |
    | `boolean` | `contains(java.lang.String fileName)` Checks whether a file is stored in the storage or not. |
    | `double` | `deleteFile(File file)` Removes a file from the storage. |
    | `File` | `deleteFile(java.lang.String fileName)` Removes a file from the storage. |
    | `double` | `deleteFile(java.lang.String fileName, File file)` Removes a file from the storage. |
    | `double` | `getAvailableSpace()` Gets the available space on this storage in MByte. |
    | `double` | `getCapacity()` Gets the total capacity of the storage in MByte. |
    | `double` | `getCurrentSize()` Gets the current size of the storage in MByte. |
    | `File` | `getFile(java.lang.String fileName)` Gets the file with the specified name. |
    | `java.util.List<java.lang.String>` | `getFileNameList()` Gets the list of file names located on this storage. |
    | `double` | `getMaxTransferRate()` Gets the maximum transfer rate of the storage in MByte/sec. |
    | `java.lang.String` | `getName()` Gets the name of the storage. |
    | `int` | `getNumStoredFile()` Gets the number of files stored on this storage. |
    | `boolean` | `hasPotentialAvailableSpace(int fileSize)` Checks whether there is enough space on the storage for a certain file. |
    | `boolean` | `isFull()` Checks if the storage is full or not. |
    | `boolean` | `renameFile(File file, java.lang.String newName)` Renames a file on the storage. |
    | `boolean` | `reserveSpace(int fileSize)` Makes a reservation of the space on the storage to store a file. |
    | `boolean` | `setMaxTransferRate(int rate)` Sets the maximum transfer rate of this storage system in MByte/sec. |

- - ### Method Detail


    - #### getName

      ```
      java.lang.String getName()
      ```

      Gets the name of the storage.

      Returns:
      :   the name of this storage


    - #### getCapacity

      ```
      double getCapacity()
      ```

      Gets the total capacity of the storage in MByte.

      Returns:
      :   the capacity of the storage in MB


    - #### getCurrentSize

      ```
      double getCurrentSize()
      ```

      Gets the current size of the storage in MByte.

      Returns:
      :   the current size of the storage in MB


    - #### getMaxTransferRate

      ```
      double getMaxTransferRate()
      ```

      Gets the maximum transfer rate of the storage in MByte/sec.

      Returns:
      :   the maximum transfer rate in MB/sec


    - #### getAvailableSpace

      ```
      double getAvailableSpace()
      ```

      Gets the available space on this storage in MByte.

      Returns:
      :   the available space in MB


    - #### setMaxTransferRate

      ```
      boolean setMaxTransferRate(int rate)
      ```

      Sets the maximum transfer rate of this storage system in MByte/sec.

      Parameters:
      :   `rate` - the maximum transfer rate in MB/sec

      Returns:
      :   true if the setting succeeded, false otherwise


    - #### isFull

      ```
      boolean isFull()
      ```

      Checks if the storage is full or not.

      Returns:
      :   true if the storage is full, false otherwise


    - #### getNumStoredFile

      ```
      int getNumStoredFile()
      ```

      Gets the number of files stored on this storage.

      Returns:
      :   the number of stored files


    - #### reserveSpace

      ```
      boolean reserveSpace(int fileSize)
      ```

      Makes a reservation of the space on the storage to store a file.

      Parameters:
      :   `fileSize` - the size to be reserved in MB

      Returns:
      :   true if reservation succeeded, false otherwise


    - #### addReservedFile

      ```
      double addReservedFile(File file)
      ```

      Adds a file for which the space has already been reserved. The time taken (in seconds) for
      adding the specified file can also be found using
      `gridsim.datagrid.File#getTransactionTime()`.

      Parameters:
      :   `file` - the file to be added

      Returns:
      :   the time (in seconds) required to add the file


    - #### hasPotentialAvailableSpace

      ```
      boolean hasPotentialAvailableSpace(int fileSize)
      ```

      Checks whether there is enough space on the storage for a certain file.

      Parameters:
      :   `fileSize` - a FileAttribute object to compare to

      Returns:
      :   true if enough space available, false otherwise


    - #### getFile

      ```
      File getFile(java.lang.String fileName)
      ```

      Gets the file with the specified name. The time taken (in seconds) for getting the specified
      file can also be found using `gridsim.datagrid.File#getTransactionTime()`.

      Parameters:
      :   `fileName` - the name of the needed file

      Returns:
      :   the file with the specified filename


    - #### getFileNameList

      ```
      java.util.List<java.lang.String> getFileNameList()
      ```

      Gets the list of file names located on this storage.

      Returns:
      :   a List of file names


    - #### addFile

      ```
      double addFile(File file)
      ```

      Adds a file to the storage. The time taken (in seconds) for adding the specified file can
      also be found using `gridsim.datagrid.File#getTransactionTime()`.

      Parameters:
      :   `file` - the file to be added

      Returns:
      :   the time taken (in seconds) for adding the specified file


    - #### addFile

      ```
      double addFile(java.util.List<File> list)
      ```

      Adds a set of files to the storage. The time taken (in seconds) for adding each file can also
      be found using `gridsim.datagrid.File#getTransactionTime()`.

      Parameters:
      :   `list` - the files to be added

      Returns:
      :   the time taken (in seconds) for adding the specified files


    - #### deleteFile

      ```
      File deleteFile(java.lang.String fileName)
      ```

      Removes a file from the storage. The time taken (in seconds) for deleting the specified file
      can be found using `gridsim.datagrid.File#getTransactionTime()`.

      Parameters:
      :   `fileName` - the name of the file to be removed

      Returns:
      :   the deleted file.


    - #### deleteFile

      ```
      double deleteFile(java.lang.String fileName,
                      File file)
      ```

      Removes a file from the storage. The time taken (in seconds) for deleting the specified file
      can also be found using `gridsim.datagrid.File#getTransactionTime()`.

      Parameters:
      :   `fileName` - the name of the file to be removed
      :   `file` - the file which is removed from the storage is returned through this parameter

      Returns:
      :   the time taken (in seconds) for deleting the specified file


    - #### deleteFile

      ```
      double deleteFile(File file)
      ```

      Removes a file from the storage. The time taken (in seconds) for deleting the specified file
      can also be found using `gridsim.datagrid.File#getTransactionTime()`.

      Parameters:
      :   `file` - the file which is removed from the storage is returned through this parameter

      Returns:
      :   the time taken (in seconds) for deleting the specified file


    - #### contains

      ```
      boolean contains(java.lang.String fileName)
      ```

      Checks whether a file is stored in the storage or not.

      Parameters:
      :   `fileName` - the name of the file we are looking for

      Returns:
      :   true if the file is in the storage, false otherwise


    - #### contains

      ```
      boolean contains(File file)
      ```

      Checks whether a file is stored in the storage or not.

      Parameters:
      :   `file` - the file we are looking for

      Returns:
      :   true if the file is in the storage, false otherwise


    - #### renameFile

      ```
      boolean renameFile(File file,
                       java.lang.String newName)
      ```

      Renames a file on the storage. The time taken (in seconds) for renaming the specified file
      can also be found using `gridsim.datagrid.File#getTransactionTime()`.

      Parameters:
      :   `file` - the file we would like to rename
      :   `newName` - the new name of the file

      Returns:
      :   true if the renaming succeeded, false otherwise


- Overview
- Package
- Class
- Use
- Tree
- Deprecated
- Index
- Help

- Prev Class
- Next Class

- Frames
- No Frames

- All Classes

- Summary:
- Nested |
- Field |
- Constr |
- Method

- Detail:
- Field |
- Constr |
- Method
